# Supplementary material for: Experimental Pathogenicity of H9N2 Avian Influenza Viruses Harboring a Tri-Basic Hemagglutinin Cleavage Site in Sonali and Broiler Chickens
Source: Viruses. 2023 Feb 7;15(2):461. doi: 10.3390/v15020461 (PMC9967266; doi:10.3390/v15020461)
Supplement: Supplementary file 1 [file viruses-15-00461-s001.zip › Supplemental Figure S1.pdf]

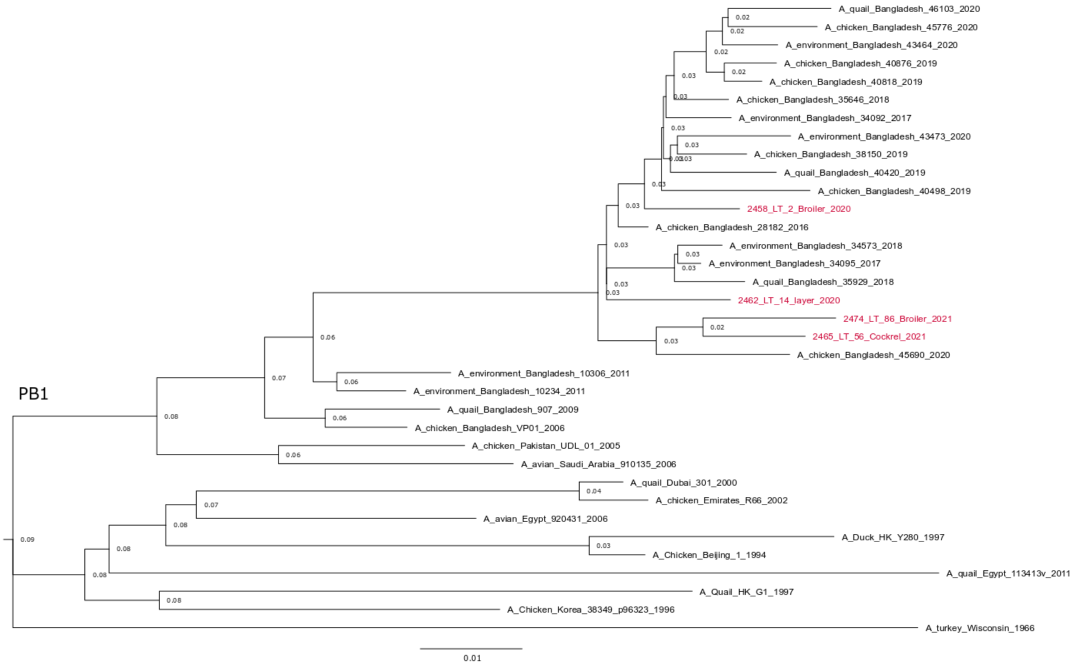
PB1
A_quail_Bangladesh_46103_2020
A_chicken_Bangladesh_45776_2020
A_environment_Bangladesh_43464_2020
A_chicken_Bangladesh_40876_2019
A_chicken_Bangladesh_40818_2019
A_chicken_Bangladesh_35646_2018
A_environment_Bangladesh_34092_2017
A_environment_Bangladesh_43473_2020
A_chicken_Bangladesh_38150_2019
A_quail_Bangladesh_40420_2019
A_chicken_Bangladesh_40498_2019
2458_LT_2_Broiler_2020
A_chicken_Bangladesh_28182_2016
A_environment_Bangladesh_34573_2018
A_environment_Bangladesh_34095_2017
A_quail_Bangladesh_35929_2018
2462_LT_14_layer_2020
2474_LT_86_Broiler_2021
2465_LT_56_Cockrel_2021
A_chicken_Bangladesh_45690_2020
A_environment_Bangladesh_10306_2011
A_environment_Bangladesh_10234_2011
A_quail_Bangladesh_907_2009
A_chicken_Bangladesh_VP01_2006
A_chicken_Pakistan_UDL_01_2005
A_avian_Saudi_Arabia_910135_2006
A_quail_Dubai_301_2000
A_chicken_Emirates_R66_2002
A_avian_Egypt_920431_2006
A_Duck_HK_Y280_1997
A_Chicken_Beijing_1_1994
A_quail_Egypt_113413v_2011
A_Quail_HK_G1_1997
A_Chicken_Korea_38349_p96323_1996
A_turkey_Wisconsin_1966
0.01

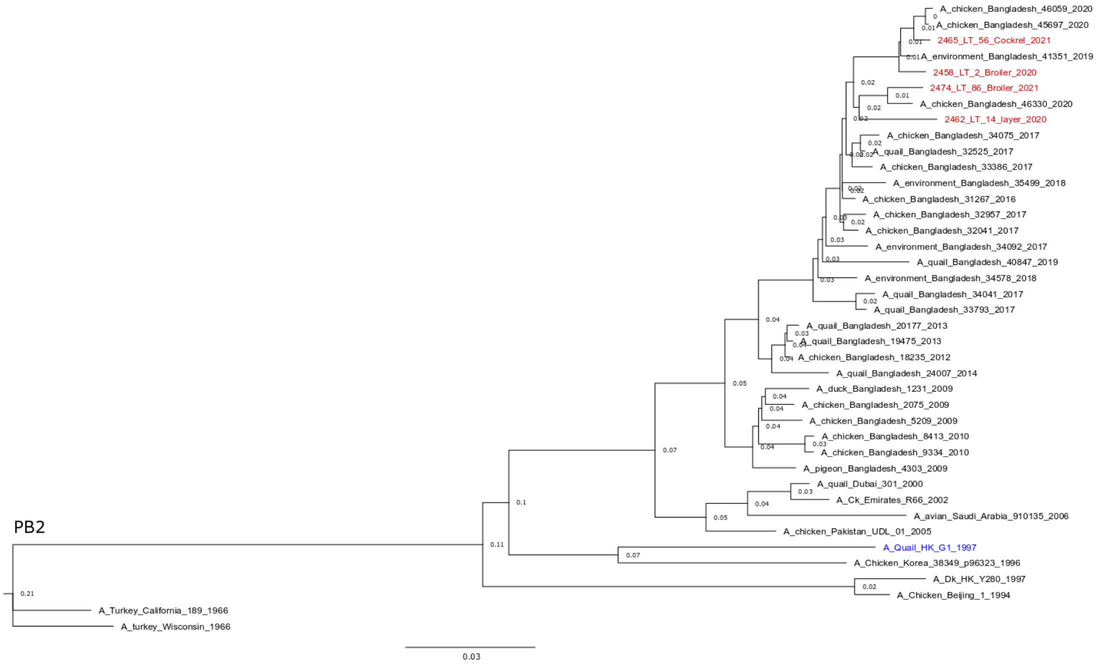
PB2
A_chicken_Bangladesh_46059_2020
A_chicken_Bangladesh_45697_2020
2465_LT_56_Cockrel_2021
A_environment_Bangladesh_41351_2019
2458_LT_2_Broiler_2020
2474_LT_86_Broiler_2021
A_chicken_Bangladesh_46330_2020
2462_LT_14_layer_2020
A_chicken_Bangladesh_34075_2017
A_quail_Bangladesh_32525_2017
A_chicken_Bangladesh_33386_2017
A_environment_Bangladesh_35499_2018
A_chicken_Bangladesh_31267_2016
A_chicken_Bangladesh_32957_2017
A_chicken_Bangladesh_32041_2017
A_environment_Bangladesh_34092_2017
A_quail_Bangladesh_40847_2019
A_environment_Bangladesh_34578_2018
A_quail_Bangladesh_34041_2017
A_quail_Bangladesh_33793_2017
A_quail_Bangladesh_20177_2013
A_quail_Bangladesh_19475_2013
A_chicken_Bangladesh_18235_2012
A_quail_Bangladesh_24007_2014
A_duck_Bangladesh_1231_2009
A_chicken_Bangladesh_2075_2009
A_chicken_Bangladesh_5209_2009
A_chicken_Bangladesh_8413_2010
A_chicken_Bangladesh_9334_2010
A_pigeon_Bangladesh_4303_2009
A_quail_Dubai_301_2000
A_Ck_Emirates_R66_2002
A_avian_Saudi_Arabia_910135_2006
A_chicken_Pakistan_UDL_01_2005
A_Quail_HK_G1_1997
A_Chicken_Korea_38349_p96323_1996
A_Dk_HK_Y280_1997
A_Chicken_Beijing_1_1994
A_Turkey_California_189_1966
A_turkey_Wisconsin_1966
0.03

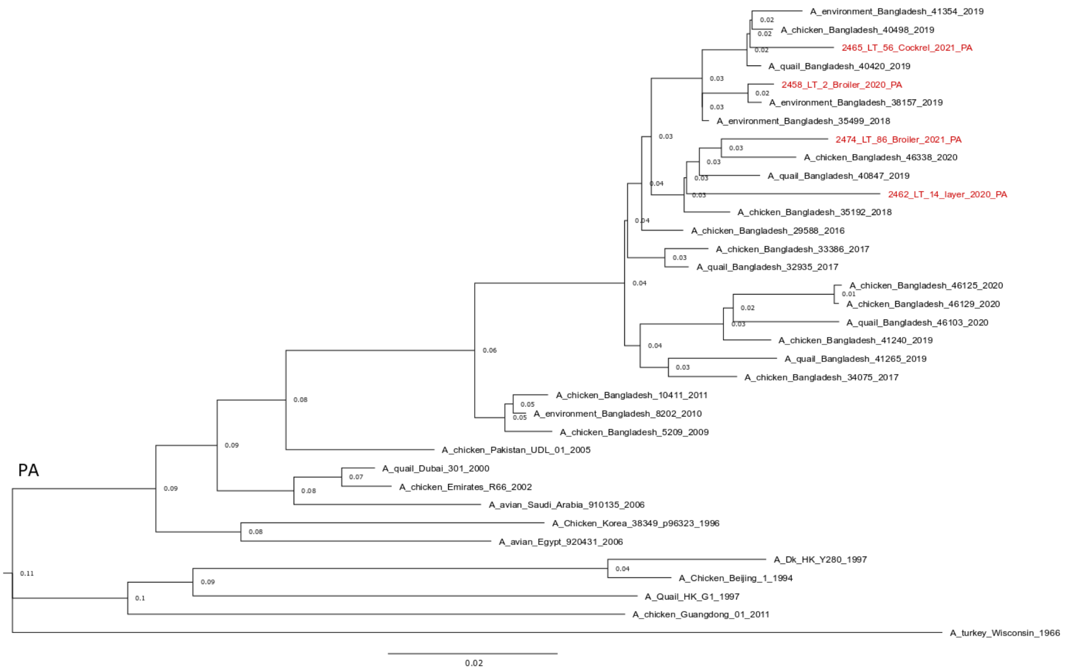
PA
A_environment_Bangladesh_41354_2019
A_chicken_Bangladesh_40498_2019
2465_LT_56_Cockrel_2021_PA
A_quail_Bangladesh_40420_2019
2458_LT_2_Broiler_2020_PA
A_environment_Bangladesh_38157_2019
A_environment_Bangladesh_35499_2018
2474_LT_86_Broiler_2021_PA
A_chicken_Bangladesh_46338_2020
A_quail_Bangladesh_40847_2019
2462_LT_14_layer_2020_PA
A_chicken_Bangladesh_35192_2018
A_chicken_Bangladesh_29588_2016
A_chicken_Bangladesh_33386_2017
A_quail_Bangladesh_32935_2017
A_chicken_Bangladesh_46125_2020
A_chicken_Bangladesh_46129_2020
A_quail_Bangladesh_46103_2020
A_chicken_Bangladesh_41240_2019
A_quail_Bangladesh_41265_2019
A_chicken_Bangladesh_34075_2017
A_chicken_Bangladesh_10411_2011
A_environment_Bangladesh_8202_2010
A_chicken_Bangladesh_5209_2009
A_chicken_Pakistan_UDL_01_2005
A_quail_Dubai_301_2000
A_chicken_Emirates_R66_2002
A_avian_Saudi_Arabia_910135_2006
A_Chicken_Korea_38349_p96323_1996
A_avian_Egypt_920431_2006
A_Dk_HK_Y280_1997
A_Chicken_Beijing_1_1994
A_Quail_HK_G1_1997
A_chicken_Guangdong_01_2011
A_turkey_Wisconsin_1966
0.02

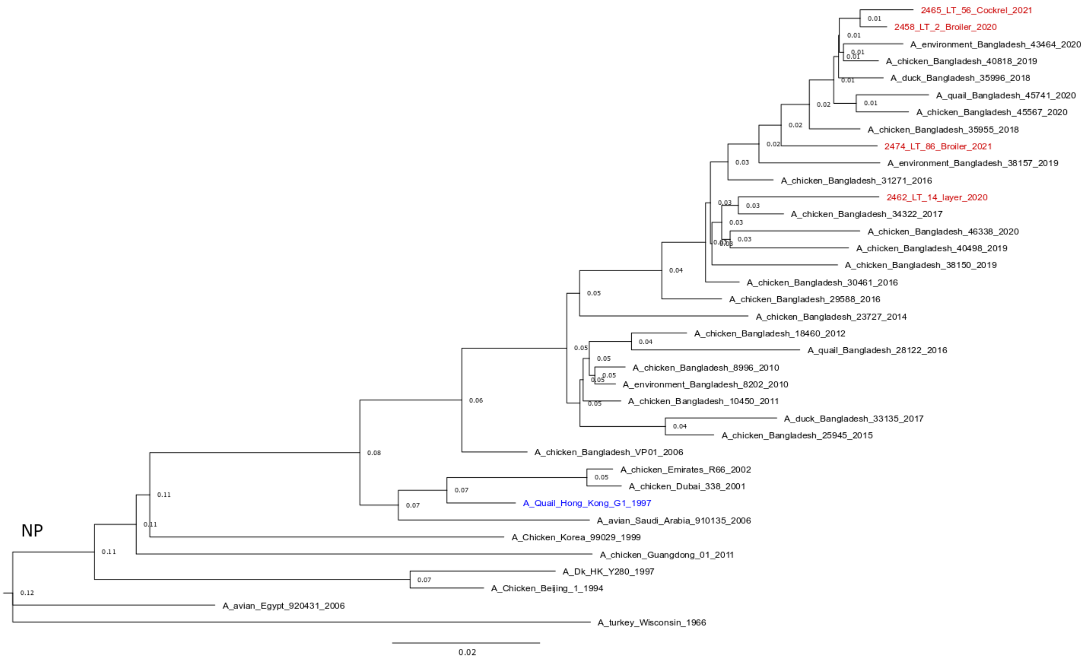
NP
2465_LT_56_Cockrel_2021
2458_LT_2_Broiler_2020
A_environment_Bangladesh_43464_2020
A_chicken_Bangladesh_40818_2019
A_duck_Bangladesh_35996_2018
A_quail_Bangladesh_45741_2020
A_chicken_Bangladesh_45567_2020
A_chicken_Bangladesh_35955_2018
2474_LT_86_Broiler_2021
A_environment_Bangladesh_38157_2019
A_chicken_Bangladesh_31271_2016
2462_LT_14_layer_2020
A_chicken_Bangladesh_34322_2017
A_chicken_Bangladesh_46338_2020
A_chicken_Bangladesh_40498_2019
A_chicken_Bangladesh_38150_2019
A_chicken_Bangladesh_30461_2016
A_chicken_Bangladesh_29588_2016
A_chicken_Bangladesh_23727_2014
A_chicken_Bangladesh_18460_2012
A_quail_Bangladesh_28122_2016
A_chicken_Bangladesh_8996_2010
A_environment_Bangladesh_8202_2010
A_chicken_Bangladesh_10450_2011
A_duck_Bangladesh_33135_2017
A_chicken_Bangladesh_25945_2015
A_chicken_Bangladesh_VP01_2006
A_chicken_Emirates_R66_2002
A_chicken_Dubai_338_2001
A_Quail_Hong_Kong_G1_1997
A_avian_Saudi_Arabia_910135_2006
A_Chicken_Korea_99029_1999
A_chicken_Guangdong_01_2011
A_Dk_HK_Y280_1997
A_Chicken_Beijing_1_1994
A_avian_Egypt_920431_2006
A_turkey_Wisconsin_1966
0.02

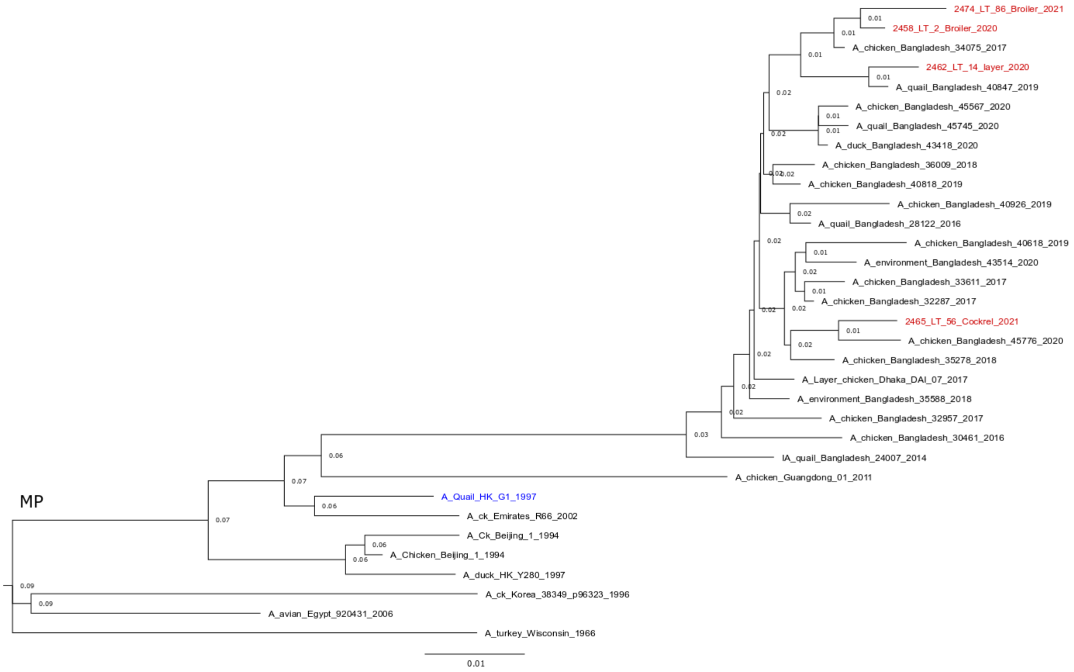

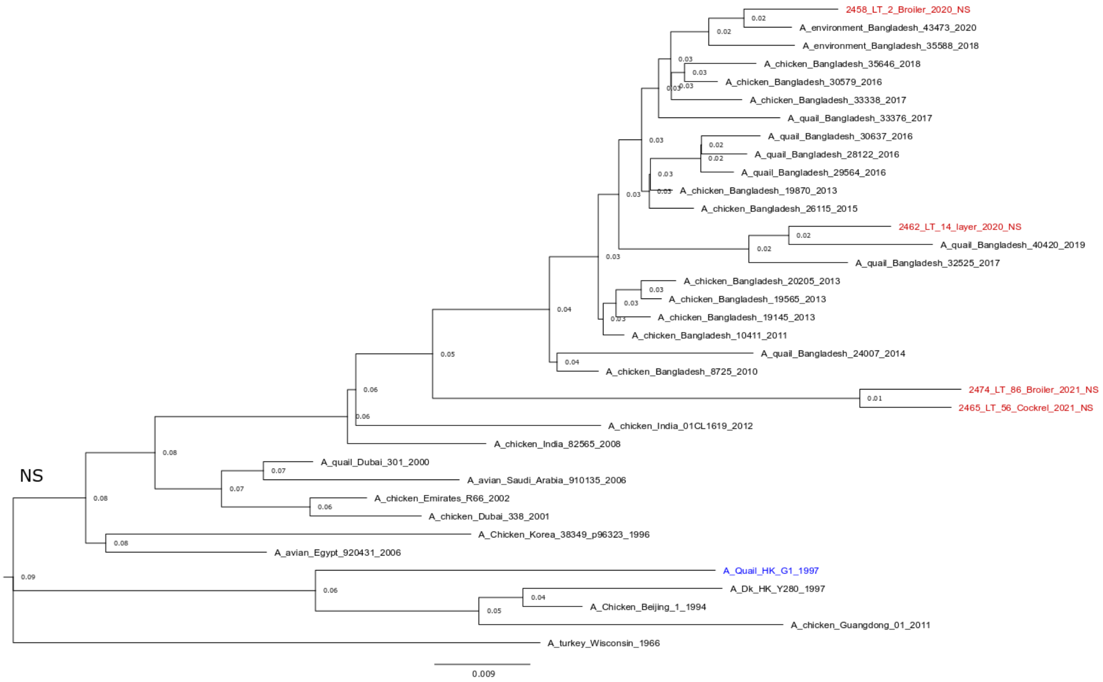

Supplemental Figure S1: Phylogenetic tree for PB1, PB2, PA, NP, MP, NS genes of recent H9N2 isolates of Bangladesh. The tree was built using neighbor joining method and Jukes-Cantor substitution model incorporated in the MAFFT package with the confidence intervals estimated by applying 1000 bootstrap algorithm. Red color taxa with circle indicate studied Bangladeshi isolates. Blue color taxa represent G1 reference strain.
