# Supplementary material for: Experimental Pathogenicity of H9N2 Avian Influenza Viruses Harboring a Tri-Basic Hemagglutinin Cleavage Site in Sonali and Broiler Chickens
Source: Viruses. 2023 Feb 7;15(2):461. doi: 10.3390/v15020461 (PMC9967266; doi:10.3390/v15020461)
Supplement: Supplementary file 1 [file viruses-15-00461-s001.zip › Supplemental Figure S2.pdf]

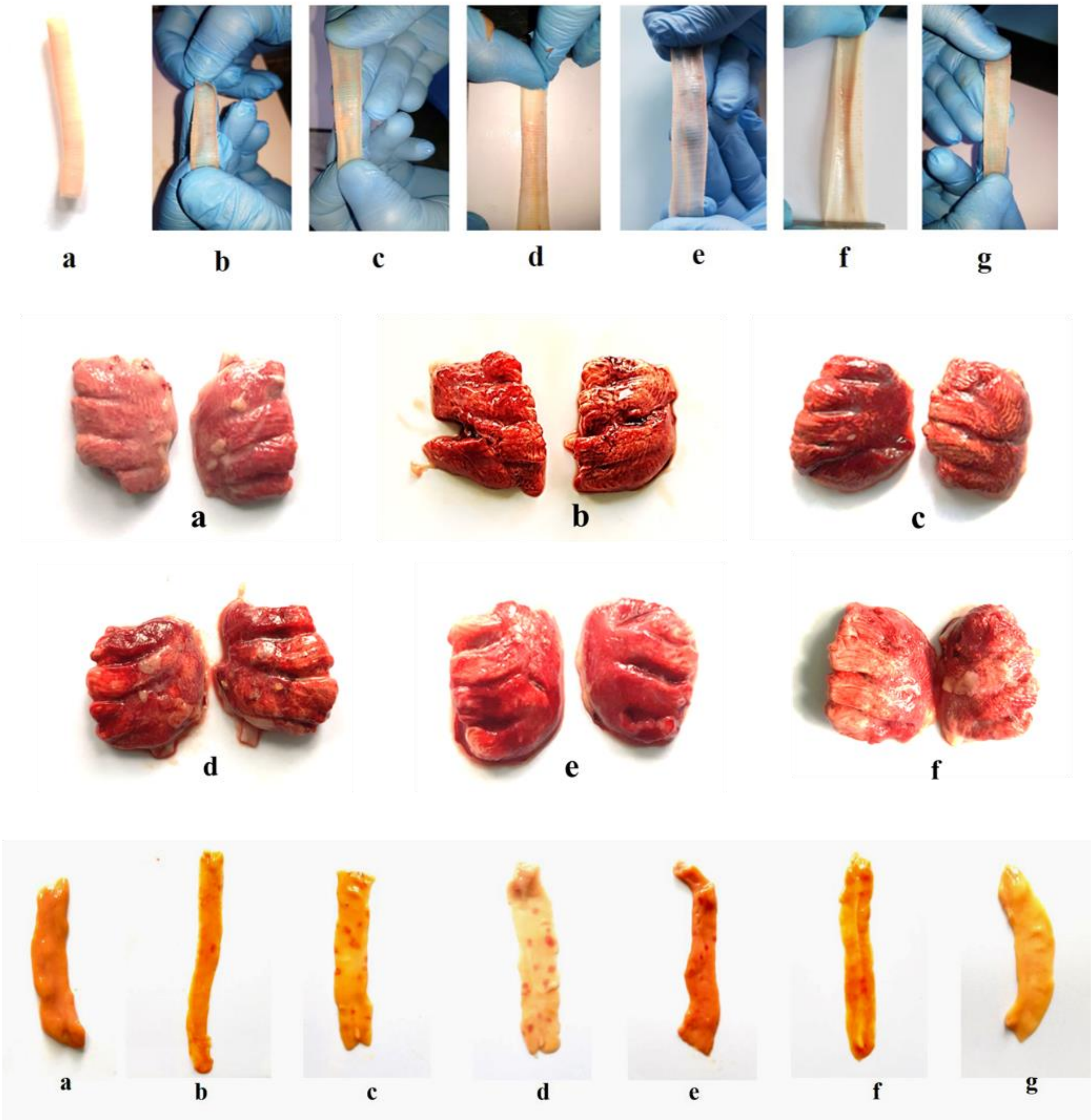

Supplemental Figure S2: Gross pathological changes observed at different post days infection (dpi) of H9N2 infected Sonali chickens. (a) indicates control bird and (b-g) indicates 1, 3, 5, 7, 10, 15 dpi, respectively. Upper panel: macroscopic changes in the trachea: (a) normal morphology of trachea, (b-g) slight hemorrhages along the tracheal ring. Middle panel: changes in the lungs: (a) lungs showing fresh pink morphology, (b-f) mild to moderate progressive congestion and fibrin deposition up to 7 dpi in the lungs. The lungs at 10 dpi are started to look somewhat normal. Lower panel: changes in the intestine: (a) normal intestinal morphology, (b-g) mild to moderate progressive petechial hemorrhages up to 7 dpi while from 10 dpi intestine started appear normal.
