# Supplementary material for: Experimental Pathogenicity of H9N2 Avian Influenza Viruses Harboring a Tri-Basic Hemagglutinin Cleavage Site in Sonali and Broiler Chickens
Source: Viruses. 2023 Feb 7;15(2):461. doi: 10.3390/v15020461 (PMC9967266; doi:10.3390/v15020461)
Supplement: Supplementary file 1 [file viruses-15-00461-s001.zip › Supplemental Figure S3.pdf]

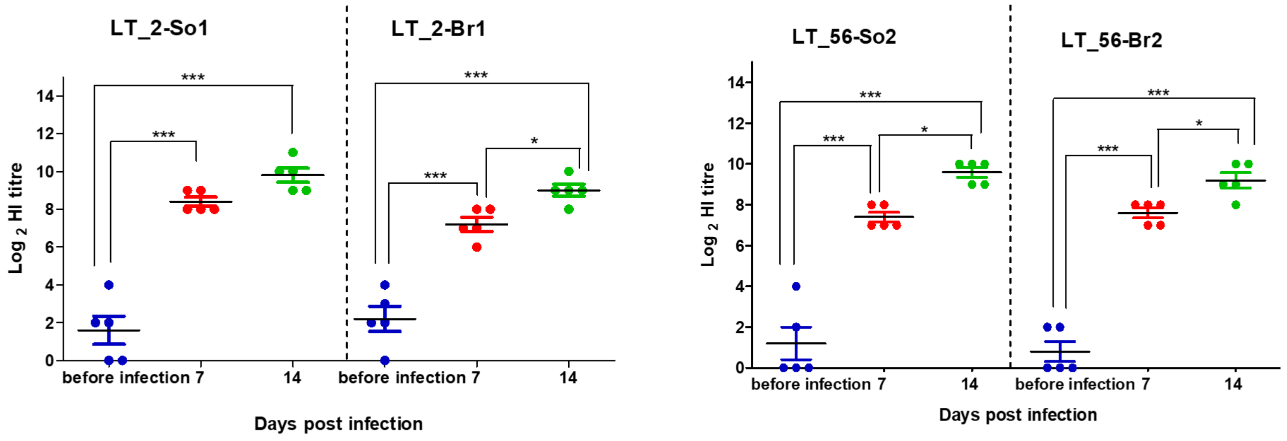

Supplemental Figure S3: Seroconversion of infected Sonali and broiler after getting 10^6 EID_{50}/ml of each virus. Level of significance are calculated through One-way ANOVA with Tukey's multiple comparison test and shown as asterisk mark.
