# Supplementary material for: Experimental Pathogenicity of H9N2 Avian Influenza Viruses Harboring a Tri-Basic Hemagglutinin Cleavage Site in Sonali and Broiler Chickens
Source: Viruses. 2023 Feb 7;15(2):461. doi: 10.3390/v15020461 (PMC9967266; doi:10.3390/v15020461)
Supplement: Supplementary file 1 [file viruses-15-00461-s001.zip › Supplemental Tables.pdf]

Supplemental Tables

Supplemental Table S1. Summary information of AIV H9 isolates from chickens characterized in this study

Sample ID/ Isolate No.	Collection date	Bird type	Age	Flock size	Vaccination	Clinical signs	Mortality	Major necropsy findings
LT-2	11.03.2020	Broiler	20 d	1200	No	respiratory distress, diarrhea	2.5%	Hemorrhages in muscle congestion in the trachea and lung; ascites; hydropericardium
LT-14	03.06.2020	Layer	32w	3500	No	respiratory distress, diarrhea, off feed	3.6%	Congestion in trachea and lung; highly fragile liver, hemorrhages in cecal tonsil
LT-56	18.01.2021	Cockrel	45 d	1500	No	respiratory distress, ruffled feather,	7.3%	Congestion in the trachea and lungs; hemorrhages in the pancreas and intestine
LT-86	16.06.2021	Broiler	28 d	800	No	respiratory distress, depression and off feed	5.8%	Congestion in the trachea, lung, liver, intestine and mesentery; mottled spleen

Supplementary Table S2: GISAID accession numbers of AIV H9 isolates from chickens characterized in this study

Isolate ID	Bird type	Genes	Accession No.
A/chicken/Bangladesh/2458-LT2/2020	Broiler	PB2	EPI2187410
		PB1	EPI2187411
		PA	EPI2187412
		HA	EPI2187413
		NP	EPI2187414
		NA	EPI2187415
		MP	EPI2187416
		NS	EPI2187417
A/chicken/Bangladesh/2462-LT14/2020	Layer	PB2	EPI2187418
		PB1	EPI2187419
		PA	EPI2187420
		HA	EPI2187421
		NP	EPI2187422
		NA	EPI2187423
		MP	EPI2187424
		NS	EPI2187425
A/chicken/Bangladesh/2465-LT56/2021	Cockrel	PB2	EPI2187426
		PB1	EPI2187427
		PA	EPI2187428
		HA	EPI2187429
		NP	EPI2187430
		NA	EPI2187431
		MP	EPI2187432
		NS	EPI2187433
A/chicken/Bangladesh/2474-LT86/2021	Broiler	PB2	EPI2187434
		PB1	EPI2187435
		PA	EPI2187436
		HA	EPI2187437
		NP	EPI2187438
		NA	EPI2187439
		MP	EPI2187440
		NS	EPI2187441

Supplemental Table S3: Major amino acid changes of HA and NA protein of studied four H9N2 viruses from Bangladesh in comparison to G1 reference strain.

**Name of the isolates**	**Proteins**	**AF156378.1 _A/Quail/HK/G1/1997***			
		**% Amino acid identity**	**Number of mutations**	**List of mutations**	**Functions related to the mutations**
A/chicken/Bangladesh/2458-LT2/2020 _15187184	HA	90.239	53	S5T, I7M, I9T, V12L, V13I, A15T, N17L, E54D, S63N, T72K, T74N, I75V, V79I, L87S, E90G, S101P, A126S, I147T, R149K, A150S, G153N, S158N, S166N, G167S, F168L, Y176F, S183D, Y196D, E198A, I204T, N206T, N218D, V224L, D233G, Q235I, D239N, V244I, G271E, K282N, T313N, V318I, R319G, V327I, R335K, S337K, M372I, R376K, D377I, I383V, K461R, R498K, R501Q, K510R	- binding small ligand(s)<br>- viral oligomerization interfaces<br>- antibody recognition sites<br>- antigenic drift / escape mutant<br>- involved in a T-cell epitope presented by MHC molecules<br>- host cell receptor binding<br>- virulence and host specificity shift<br>- virulence, antigenic drift / escape mutant<br>- host specificity shift<br>-antigenic drift / escape mutant and virulence.<br>- create or removes a potential N-glycosylation site
A/chicken/Bangladesh/2462-LT14/2020_15187185		90.239		S5T, I7M, I9T, V12L, V13I, A15T, N17I, E54D, S63N, T74N, I75V, V79I, L87S, E90G, S101P, A126S, I147T, R149K, A150S, G153D, S158N, S166N, G167S, F168L, Y176F, S183D, Y196D, E198T, I204T, N206T, N218D, V224L, D233G, Q235I, D239N, V244I, G271E, D280N, K282N, T313N, V318I, R319G, V327I, R335K, S337K, M372I, R376K, D377V, I383V, K461R, R498K, R501Q, K510R	
A/chicken/Bangladesh/2465-LT56/2021 _15187186		89.871	55	S5T, I7M, I9T, V12L, V13I, A15T, N17L, E54D, S63N, H66R, T72K, T74N, I75V, V79I, L87S,	

				E90G, S101P, A126S, I147T, R149K, A150S, G153D, S158N, S166N, G167S, F168L, Y176F, S183D, Y196D, E198A, I204T, N206T, N218D, V224L, D233G, Q235I, D239N, V244I, G271E, K282N, V287A, I306V, T313N, R319G, V327I, R335K, S337K, M372I, R376K, D377I, I383V, K461R, R498K, R501Q, K510R	
A/chicken/Bangladesh/2474-LT86/2021 _15187187		90.239	53	S5T, I7M, I9T, V12L, V13I, A15T, N17L, E54D, S63N, T72K, T74N, I75V, V79I, L87S, E90G, S101P, A126S, I147T, R149K, A150S, G153N, S158N, S166N, G167S, F168L, Y176F, S183D, Y196D, E198A, I204T, N206T, N218D, V224L, D233G, Q235I, D239N, V244I, G271E, K282N, T313N, V318I, R319G, V327I, R335K, S337K, M372I, R376K, D377I, I383V, K461R, R498K, R501Q, K510R	
A/chicken/Bangladesh/2458-LT2/2020 _15187184	NA	91.126	43	V13A, I15L, T19I, I20V, M24I, M33V, ins37Q, ins37T, C40S, P43S, N45I, Q47K, P53S, N68G, T70I, K73R, V79P, G123S, G125N, K141R, H153Y, N159S, R197K, M208L, R247K, K251R, L253I, R281L, V305M, S309N, V310I, S313N, N354T, N356D, K365E, S370A, T383L, I390T, D397E, I400N, S404Y, A410V, I414T	- binding small ligand(s)<br>- viral oligomerization interfaces<br>- antibody recognition sites<br>- creates or removes a new potential N-glycosylation site<br>- strong drug resistance<br>- antigenic drift / escape mutant.<br>- drug binding
A/chicken/Bangladesh/2462-LT14/2020_15187185		89.610	50	G11S, V13A, I15L, T19I, I20V, M24I, ins37H, ins37T, C40Y, T41I, P43S, N45T, Q47K, P53S, I54T, N68G, T70V, K73R, V79P, A80S, G123S,	

				G125N, K141R, H153Y, N159S, V163I, I174M, K185R, R197K, M208L, R247M, K251R, V305M, S309N, V310I, D311Y, S313N, N334D, R336K, N356D, K365Q, S370A, T383V, I390T, D397E, I400N, S404Y, A410V, G412S, I414T	
A/chicken/Bangladesh/2465-LT56/2021 _15187186		91.126	43	V13A, I15L, T19I, I20V, M24I, M33V, ins37Q, ins37T, C40Y, P43S, N45T, Q47K, P53S, N68G, T70I, K73R, K78T, V79P, G123S, G125N, K141G, H153Y, N159S, R197K, M208L, R247K, K251R, L253I, R281L, V305M, S309N, V310I, S313N, N356D, K365E, S370A, T383L, I390T, D397E, I400N, S404Y, A410V, I414T	
A/chicken/Bangladesh/2474-LT86/2021 _15187187		90.260	47	V13A, I15L, T19I, I20V, M24I, ins37Q, ins37T, C40Y, P43S, N45T, Q47K, A48T, P53S, I54T, N68G, T70I, K73R, V79P, N84S, Q91P, G123S, G125N, N139D, K141R, H153Y, N159S, K185R, R197K, M208L, R247I, K251R, L253I, V305M, A306V, S309N, S313N, N356D, S370A, V378I, G379D, T383V, I390T, D397E, I400N, S404Y, A410V, I414T	

*The comparison was based on the mutation analysis at FluSurver of GISAID platform
